# Supplementary material for: INDUCING REPRESENTATIONAL CHANGE IN THE HIPPOCAMPUS THROUGH REAL-TIME NEUROFEEDBACK
Source: bioRxiv. 2023 Dec 4:2023.12.01.569487. Preprint. [Version 1] doi: 10.1101/2023.12.01.569487 (PMC10723264; doi:10.1101/2023.12.01.569487)
Supplement: Supplement 1 [file NIHPP2023.12.01.569487V1-supplement-1.pdf]

bioRxiv preprint doi: https://doi.org/10.1101/2023.12.01.569487; this version posted December 4, 2023. The copyright holder for this preprint (which was not certified by peer review) is the author/funder, who has granted bioRxiv a license to display the preprint in perpetuity. It is made available under aCC-BY 4.0 International license.

<table>
<tr><td colspan="6">procedure</td></tr>
<tr><td colspan="4">if first neurofeedback training run of session 1</td><td rowspan="9">then</td><td>threshold=60%</td></tr>
<tr><td colspan="4">else if first neurofeedback training run of day N</td><td>threshold = last threshold from day N-1</td></tr>
<tr><td rowspan="7">else if number of successful trials</td><td><=1</td><td rowspan="7">for the previous</td><td>1 run</td><td rowspan="3">decrease threshold by 5% (min 40%)</td></tr>
<tr><td><=3</td><td>3 runs</td></tr>
<tr><td><=5</td><td>5 runs</td></tr>
<tr><td>==6</td><td>any runs</td><td>keep threshold unchanged</td></tr>
<tr><td>>=7</td><td>5 runs</td><td rowspan="3">increase threshold by 5% (max 90%)</td></tr>
<tr><td>>=9</td><td>3 runs</td></tr>
<tr><td>>=11</td><td>1 run</td></tr>
</table>

Supplementary Table 1. Staircase procedure for adaptive threshold. Trials were considered successful when 2 or more of the TRs (of 5 total feedback TRs per trial) were above the threshold.
